# Hypothesis: Entrapment of lipoprotein particles in the brain causes Alzheimer’s disease

**DOI:** 10.17879/freeneuropathology-2021-3459

**Published:** 2021-11-02

**Authors:** Delphine Boche, James AR Nicoll

**Affiliations:** 1 Clinical Neurosciences, Clinical and Experimental Science, Faculty of Medicine, University of Southampton, Southampton, United Kingdom; 2 Department of Cellular Pathology, University Hospital Southampton NHS Foundation Trust, Southampton, United Kingdom

**Keywords:** Lipoprotein particles, Extracellular matrix, Cholesterol transport, Apolipoprotein E, Alzheimer’s disease

## Abstract

We present for consideration a hypothesis that impaired movement of lipoprotein particles in the extracellular space in the brain in ageing is central to and causes all the key pathophysiological features of Alzheimer’s disease (AD). The role of lipoprotein particles is to transport cholesterol from glial cells, where it is synthesised, to neurons, which require cholesterol for synaptic plasticity. The lipoprotein particles have a cholesterol-containing hydrophobic core, in which amyloid-β (Aβ) can be solubilised. The core is surrounded by a hydrophilic surface containing apolipoprotein E (APOE) which, as neurons bear receptors for APOE, determines the destination of the particles. The problem arises because the extracellular space is a narrow cleft, barely wider than the lipoprotein particles themselves, which they have to navigate in order to perform their crucial cholesterol-transporting function. We explain how lipoprotein particles could become trapped in the ageing extracellular matrix and that this primary abnormality results in reduced delivery of cholesterol to neurons leading to impaired synaptic plasticity, crucial for learning and memory. It can also explain extracellular Aβ accumulation, to which a microglial response generates a neurotoxic reaction, and intraneuronal tau aggregation, each of which exacerbate the problem. All these players have been known for many years to be important in Alzheimer’s pathogenesis but a single unifying mechanism to explain how they are linked has been lacking. This proposed mechanism, with entrapment of lipoproteins particles as key to the development of AD, can explain the failure of so many clinical trials and points out new directions to be taken.

## Genetics shows the overriding importance of apolipoprotein E

In a complex multifaceted disease process, such as Alzheimer’s disease (AD), genetic factors are important in highlighting key elements relevant to the onset of the disease. By far the major genetic risk factor for AD, in terms of scale of effect, is the apolipoprotein E genotype (gene: *APOE,* protein: APOE). There are 3 common *APOE* gene alleles (ε2, ε3 and ε4); as each person inherits one allele from either parent there is a total of 6 *APOE* genotypes (ε2/ε2, ε2/ε3, ε3/ε3, ε3/ε4 and ε4/ε4). The *APOE* ε4 allele carriage rate (i.e. the proportion of people in a population who possess one or two ε4 alleles) is typically about 25% of people of European ancestry [1], but varies considerably around the world among different populations [2]. A single copy of the *APOE* ε4 allele confers a two to fourfold increased risk of developing AD, with ε4 homozygotes at fourteen times increased risk of developing AD, whereas the less common ε2 allele confers relative protection, approximately halving the risk [3]. The effect is so substantial that a person who is ε4 homozygous and lives to 85 years of age has a lifetime risk of AD of more than 50%, comparable to the risk associated with BRCA1 mutation in breast cancer [4]. On the other hand, the protective effect of *APOE* ε2 is so substantial that ε2 homozygotes have a very low likelihood of developing AD; ε2 homozygotes have a 87% lower odds ratio than ε3 homozygotes and a 99.6% lower odds ratio than ε4 homozygotes [5]. The *APOE* gene polymorphism is lacking from non-human primates [6, 7] although, interestingly, Rhesus monkeys which have an ε4-like *APOE* sequence develop Aβ plaques as they age [8]. The fact that there are more than 200 animal models of AD highlights the difficulty in mimicking the complexity of the human disease in experimental animals. A complete understanding of the pathophysiology of AD ideally would explain all aspects of the human disease, and in particular incorporate the role of the APOE protein; how it causes and interacts with the accumulation of amyloid-β (Aβ) and tau proteins, the glial cell activity and most importantly, the neuronal and synaptic dysfunction and loss.

## The function of APOE is to deliver cholesterol, packaged in lipoprotein particles, to neurons

APOE is the principal cholesterol carrier in the brain, acting as a detergent with hydrophobic and hydrophilic moieties. Its main role is to solubilise cholesterol and other lipids and lipid-soluble substances to enable them to be transported in the aqueous extracellular environment of the brain. The importance of cholesterol to the brain is highlighted by the fact that 25% of the body’s cholesterol is contained within the brain, despite the brain representing only 2% of the body weight [9]. Cholesterol forms about 30% of the lipid bilayer of the membrane of all cells and is important in maintaining membrane fluidity. In the brain, the cell membrane is essential for conducting the action potential and for communication between neurons at synapses. However, despite its importance to neuronal function, neurons do not synthesise their own cholesterol, but rely on cholesterol which is synthesised by glial cells and then transported to neurons [10]. Delivery of cholesterol to neurons is in particular demand when the neuron changes, as in synaptic plasticity which underpins learning and memory which are particularly affected in AD [11]. Of note, genome-wide association studies have highlighted polymorphisms in genes in addition to *APOE* that are involved in cholesterol handling *(CLU, PICALM, BIN1* and *ABCA7)* as risk factors for AD [12, 13], supporting an important role for cholesterol handling in the disease mechanism [14, 15].

APOE is located in the shell of lipoprotein particles, with hydrophobic substances including cholesterol, which is loaded onto the lipoprotein particle by the enzyme ABCA1, being transported in the core [16]. The destination of the lipoprotein particles is determined by the receptors for the proteins on the surface of the lipoprotein shell. APOE, on the outer surface of the lipoprotein particles, binds to receptors of the low-density lipoprotein (LDL) receptor family (principally LRP1/APOE receptor) present on neuronal cell membranes and, by this mechanism, the cholesterol is internalised within the neurons. Some CNS lipoprotein particles also bear APOJ (clusterin) on their surface; ependymal cells, but not neurons or other glia, bear APOJ receptors so it is unlikely to be relevant for cholesterol delivery to neurons [17]. However, it is notable that in genome-wide association studies, polymorphism of APOJ/clusterin gene has also been shown to influence risk for AD [18], raising the possible importance of clearance of lipoprotein particles and cholesterol to the CSF.

Outside the brain, in the rest of the body, there are several other lipoproteins that can fulfil the function of cholesterol transport in addition to APOE, including APOA which is synthesised in the liver [19]. APOA-containing lipoprotein particles are detectable in the CSF but appear to be excluded from the brain parenchyma by the blood-brain barrier. Consequently, the cholesterol required by neurons is synthesised within the brain and delivered to neurons by APOE-containing lipoprotein particles [20] [9, 21].

## Lipoprotein particles become entrapped in the ageing extracellular matrix

The lipoprotein particles, which resemble the high-density lipoproteins (HDL) present in the bloodstream, are in the region of 11-20 nm in diameter [22]. In order to transport cholesterol to neurons, they have to travel in the extracellular space in the brain which itself measures only about 40 nm between adjacent cells [23], below the resolution of light microscopy. Small molecules can pass readily, by diffusion and potentially by active flow, through the extracellular space but diffusion even of conventional macromolecules much smaller that lipoprotein particles is hindered, particularly in pathological processes such as gliosis (activation of glial cells) [24], which occurs in association with ageing and the neurodegeneration in AD.

An additional complexity is that the extracellular space resembles a sponge, containing the extracellular matrix (ECM). In the brain, the ECM is a complex multimolecular three-dimensional structure consisting of proteoglycans/glycosaminoglycans, proteins, proteinases, and cytokines [25]. Expression of collagen IV, laminin and fibronectin is upregulated in the cerebral cortex in early AD. With ageing-related changes to the extracellular matrix, exacerbated by other risk factors for AD (hypertension, diabetes, obesity, inflammation and physical inactivity), the narrow extracellular space likely becomes compromised (‘fibrosed’), impeding the movement of, and trapping, lipoprotein particles in between cells.

**Figure 1:**
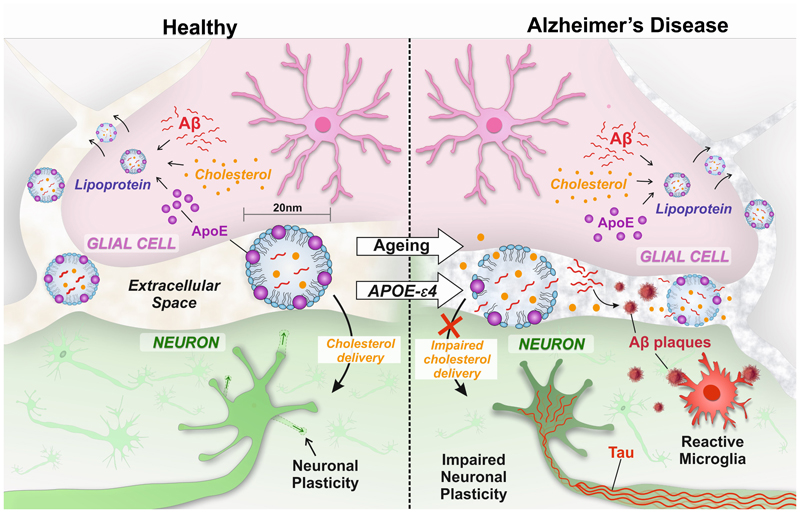
Entrapment of lipoprotein particles in the brain causes Alzheimer’s disease. Lipoprotein particles transport cholesterol from glial cells, where it is synthesised, to neurons which require cholesterol for synaptic plasticity. The lipoprotein particles have a cholesterol-containing hydrophobic core, in which Aβ is solubilised, and a hydrophilic surface containing apolipoprotein E. Receptors for APOE on the neuronal cell membrane determine the destination of the particles. The lipoprotein particles must navigate the extracellular space to reach the neurons, a narrow cleft barely wider than the particles themselves. As the extracellular matrix ages, lipoprotein particles become trapped between cells and this primary abnormality results in reduced delivery of cholesterol to neurons leading to impaired synaptic plasticity, crucial for learning and memory. Impaired movement of lipoprotein particles in the extracellular space in the brain in ageing is central to and causes all the key pathophysiological features of Alzheimer’s disease: degeneration of entrapped lipoprotein particles releases Aβ which aggregates in the aqueous environment of the extracellular space; a microglial reaction to the Aβ results in secretion of neurotoxic substances; neuronal cholesterol deficiency causes intraneuronal tau accumulation. [Artwork by Dr Jennifer M Dewing]

Interestingly, oxidative modification of plasma lipoproteins is one of the earliest steps in the development of atherosclerosis, involving accumulation of lipoprotein particles in the vessel wall provoking inflammatory reaction [26]. In AD patients, there is evidence of greater oxidation of plasma and CSF lipoproteins [26] and this could conceivably further impair the passage of lipoprotein particles through the extracellular space in the brain. Consequently, the APOE-mediated system for transporting cholesterol and lipids from glial cells to neurons in the brain is unique, crucial for neuronal function and plasticity and vulnerable to age-related failure (Figure 1). It is proposed that this results in a number of consequences leading to the key features of AD pathophysiology as follows.

## Entrapment of lipoprotein particles can explain the key features of Alzheimer’s disease

### Aβ deposition

#### In the parenchyma as plaques

Aβ peptide is notoriously insoluble in an aqueous environment, but it is lipid soluble and is transported in the hydrophobic core of lipoprotein particles [27–30]. Therefore, trapping lipoprotein particles would immobilise Aβ in the extracellular space. As the trapped particles degrade and rupture, we propose they release Aβ peptide into the surrounding aqueous environment where it aggregates, initiating the formation of extracellular Aβ plaques. Co-localisation of APOE with Aβ plaques is consistent with the origin of the Aβ as being from APOE-containing lipoprotein particles [31]. Immunohistochemistry for Aβ on semi-thin sections (1μm) shows that diffuse plaques, usually interpreted as the earliest stage of Aβ deposition, are formed from a cluster of dot-like structures [32], and electron microscopy shows small vesicles associated with amyloid fibrils in the extracellular space [32]. In the context of the current hypothesis, it is intriguing to speculate that these might be entrapped lipoprotein particles from which the amyloid has originated.

APP transgenic mice, over-expressing the V717F human amyloid precursor protein, develop Aβ plaques as they age. Interestingly, when crossed with *APOE* knock out mice so that they lack APOE, these mice do not accumulate plaques [33]. This finding indicates that APOE is essential for the deposition of Aβ plaques, supporting the mechanism we propose.

With ageing, the primary risk factor for AD, the human brain decreases in weight and size with an overall decline in cortical cholesterol that accelerates from the age of 80 [34, 35]. This is consistent with age-associated entrapment of the lipoprotein particles impairing delivery of cholesterol to neurons and resulting in neuronal cholesterol deficiency [36]. This has further effects on Aβ as cholesterol deficiency leads to thinning of the cell membrane, shifting the site for secretase cleavage of the amyloid protein precursor (APP) from producing pre-dominantly the shorter form of Aβ (Aβ40) to the longer form (Aβ42) which is relatively even more insoluble and prone to aggregation [37, 38]. This exacerbates the effect of trapped lipoprotein particles as the Aβ42 then coalesces onto the initial plaque seeds facilitating their growth.

In physiological conditions, Aβ is suggested to act as a regulator of cholesterol homeostasis as observed in experimental models [39]. In humans, the familial forms of Alzheimer’s disease are due to an imbalance in Aβ production and are associated with increased cholesterol levels [40]. The binding of APOE to lipoprotein receptor-related protein (LRP) [41], the major APOE receptor on neurons, is also involved in the cellular uptake of cholesterol and Aβ, consistent with APOE, cholesterol and Aβ all being components of the lipoprotein particle.

#### In the blood vessel walls as cerebral amyloid angiopathy (CAA)

Aβ also colocalises with APOE in the walls of cerebral blood vessels suggesting that lipoprotein particles become trapped in the extracellular space of the vessel wall basement membrane as a consequence of age-related changes. The blood vessel dysfunction caused by the presence of CAA further compromises brain function by haemorrhage, ischaemia and paralysing autoregulation of cerebral blood flow [42].

### Impaired neuronal and synaptic function

Deficiency of neuronal cholesterol results in impaired communication between neurons at the synapse, and particularly it interferes with the alterations in neurons which underpin learning and memory (i.e. synaptic plasticity) [43–46]. Synaptic plasticity requires neuronal cell membrane to be synthesised to form and re-form synapses as they are remodelled. In neuronal cultures, the presence of glial cells enhances the formation and function of synapses and the essential factor mediating this effect has been identified as glia-derived cholesterol, delivered to the neurons by APOE-containing lipoprotein particles binding to the neuronal LDL receptors [11, 47, 48].

Interestingly, in a number of clinical studies of traumatic brain injury, the possession of *APOE* ε4 is associated with a worse outcome and severe neurologic deficits [49–51]. This seems particularly so in young people in whom neuronal plasticity after injury might otherwise be more pronounced [50]. Experimental studies in *APOE* ε4 transgenic mice confirmed impaired neuronal plasticity after brain injury [52–56]. Further evidence comes from experimental models of global cerebral ischaemia in which APOE-deficient mice have increased neuronal damage which is ameliorated by intraventricular infusion of lipoprotein particles [57]. In addition, agonists of Liver X receptors (LXR), which act as cholesterol sensors and promote lipidation of APOE by ATP-binding cassette transporter A1 (ABCA1), ameliorate neuronal injury in experimental models of trauma [58] and reverse deficits in mouse models of AD [59].

The effects of neuronal and synaptic dysfunction due to lack of cholesterol [60] would be expected to be most pronounced in neuroanatomical locations in which plasticity is greatest (i.e. hippocampus involved in memory, association cortex involved in interpretation of sensations) and less severe or absent where plasticity is least (i.e. primary motor and sensory cortex, cerebellum, spinal cord). This is consistent with the clinical observations that the hierarchical sequence of loss of functions over time in AD follows the distribution of neuroplasticity [61].

### Tau protein accumulation

We propose that neuronal cholesterol deficiency, resulting from the entrapment of lipoprotein particles, leads to the intracellular aggregation of tau. Tau is involved in maintaining the cytoskeletal structure of neurons and, in particular, the transport of proteins from the cell body along axons to the synapses. Accumulation of hyperphosphorylated tau occurs early in regions where the cholesterol is most in demand, that is where the rate of plasticity and synaptic remodelling is greatest (i.e. hippocampus, association cortex) and later or not at all where plasticity is least (i.e. primary motor and sensory cortex, cerebellum, spinal cord). A difficulty in trying to explain a direct link between Aβ and tau pathology in AD has been that they appear to arise in different neuroanatomical locations; tau accumulation occurs earlier in the hippocampus and associated structures, only spreading later to the cerebral neocortex, whereas Aβ accumulation occurs early in the cerebral neocortex. The hypothesis proposed here that there is not a direct link between Aβ and tau pathology, but rather that they are each driven by cholesterol deficiency, resolves this conundrum.

Direct experimental support comes from cholesterol depletion in neuronal cultures which induces tau hyperphosphorylation that can be prevented by treatment with lipoproteins and cholesterol [62]. The link between neuronal cholesterol deficiency and tau accumulation is further highlighted by the occurrence of tangles at a young age in Niemann-Pick type disease type C, a rare progressive genetic disorder characterised by the inability of the body to transport cholesterol and lipids [63–64].

Additional circumstantial evidence supporting a link between cholesterol deficiency and tangle formation potentially comes from the study of chronic traumatic encephalopathy (CTE). CTE is a condition caused by repeated blows to the head, typically in boxing and other sports, and is associated with the development of dementia with the formation of tangles as the key pathological feature [65]. It has been found that after a head injury, levels of APOE and cholesterol-containing lipoproteins in the cerebrospinal fluid plummet, just at the time when there is an increased demand for cholesterol for neuronal repair [66–68]. This relative deficiency of cholesterol, when recurrent over time with repeated blows to the head, could explain the development of tangles in CTE [51–56] [65]

Studies using cerebral organoids derived from AD patients highlight an association of tau pathology with *APOE* ε4 carriage [69].

### Glial cell dysfunction

Astrocytes and microglia have a major role in supporting neurons and activation of glial cells is a hallmark of AD. In particular, they play an important role in the cycling of lipoprotein particles, scavenging lipid debris from degenerating neuron/synapses, lipidation the particles and releasing them for transport and uptake by neurons [70]. In *APOE* ε4 carriers, the brain is relatively deficient in APOE and the lipoprotein particles are smaller [71], more prone to aggregate and carry less cholesterol, rendering them particularly vulnerable to the effects of age-related cholesterol deficiency. Human induced pluripotent cell (iPSC)-derived astrocytes from ε4 homozygotes produce lipoprotein particles that are smaller, carry less cholesterol and support neurons less well in terms of viability and expression of synaptic proteins compared with those from ε3 carriers [72].

Microglia are the immune cells of the brain and are markedly activated in AD [73, 74]. Many of the genes identified in genome-wide association studies are expressed by microglia, indicating that they play an important role in the development and/or progression of the disease [75]. The presence of extracellular Aβ expelled by trapped and degraded lipoprotein particles is putatively recognised by the microglial pattern recognition receptors (PPRs) evolved to detect molecular patterns associated with the bacterial cell walls. The consequent pro-inflammatory state provoked by this response causes release of cytotoxic substance evolved to destroy invading micro-organisms, which inadvertently has a harmful effect on neurons, compounding the effects of cholesterol deficiency [73, 76].

### Weaknesses/limitations of the hypothesis

The ideas presented here form a hypothesis to explain the development of AD which is coherent and explains many facets of the disease. It is important to emphasise that a hypothesis is also an extrapolation of what is currently known and so some of the statements above are not firmly established but are potentially controversial and remain to be explored in the testing of the hypothesis. Potential thorns in the side of this hypothesis include:

- Localisation of Aβ within APOE-containing lipoprotein particles has not been directly demonstrated in the human brain. This needs to be explored and represents an important gap in our knowledge. Available technology is a limitation because the lipoprotein particles are below the resolution of light/confocal microscopy but are potentially amenable to study by novel 3D electron microscopy methods combined with multilabel immunostaining.
- Direct evidence that lipoprotein particles become trapped in the extracellular space, disintegrate and release Aβ is currently lacking.
- Studies of immunohistochemistry for Aβ and APOE identify APOE in some, but not all plaques. APOE seems to be present particularly in cored plaques, which are interpreted as later stage plaques, rather than early diffuse plaques as might be predicted from the hypothesis. The presence of APOE in later stage plaques could possibly reflect involvement of APOE-containing lipoprotein particles in attempted removal of Aβ, in addition to a role in plaque formation.
- Evidence that high peripheral cholesterol levels are associated with increased risk of AD and that statins, which reduce circulating cholesterol by inhibiting the cholesterol-synthesising enzyme HMG-CoA reductase, may reduce risk for AD [77] might seem to contradict the hypothesis presented here. It seems that statins do reduce cholesterol levels in the brain [78]. However, it may be that the relative amounts of cholesterol, Aβ and APOE are important i.e., that there is sufficient cholesterol as required by neurons, sufficient APOE to solubilise the cholesterol and a sufficient volume of lipophilic core within the lipoprotein particles to transport Aβ and prevent its escape into the aqueous environment of the extracellular space where it is prone to aggregate.

## Therapeutic consequences

Over the past decades, human clinical trials of new therapies for AD have been unrelentingly disappointing. This is despite there being no shortage of endeavour and expenditure, and no lack of successful therapeutic studies in animal models of specific aspects of AD. Why is this? We suggest it is because the wrong targets have been addressed. Whereas each of the major suspects listed above (Aβ, tau, glial cells) may exacerbate the neurodegeneration and set up self-perpetuating vicious cycles, we propose they are not the initiating factor in sporadic AD, either singly or in combination. The pathophysiological scheme outlined above indicates that, according to this hypothesis, Aβ and tau are not the cause of AD but are a consequence of the primary abnormality, which is trapping of lipoprotein particles in the extracellular space resulting in disruption of the normal system of delivery of cholesterol to neurons. This implies that even if, for example, the AD brain can be completely cleared of Aβ plaques, this will not halt the neurodegenerative decline as observed first in the long-term follow up of AD patients immunised against Aβ using AN1792 [79] and also in subsequent trials. Instead, therapy for AD must be directed at the health of the extracellular space, cholesterol and APOE, lubricating the passage of cholesterol-containing lipoprotein particles.
